# Project house water: a novel interdisciplinary framework to assess the environmental and socioeconomic consequences of flood-related impacts

**DOI:** 10.1186/s12302-017-0121-1

**Published:** 2017-07-10

**Authors:** Sarah E. Crawford, Catrina Brüll nee Cofalla, Benedikt Aumeier, Markus Brinkmann, Elisa Classen, Verena Esser, Caroline Ganal, Elena Kaip, Roger Häussling, Frank Lehmkuhl, Peter Letmathe, Anne-Katrin Müller, Ilja Rabinovitch, Klaus Reicherter, Jan Schwarzbauer, Marco Schmitt, Georg Stauch, Matthias Wessling, Süleyman Yüce, Markus Hecker, Karen A. Kidd, Rolf Altenburger, Werner Brack, Holger Schüttrumpf, Henner Hollert

**Affiliations:** 1 Institute for Environmental Research, RWTH Aachen University, Worringerweg 1, 52074 Aachen, Germany; 2 Institute for Hydraulic Engineering and Water Management, RWTH Aachen University, Mies van der Rohe-Straße 17, 52074 Aachen, Germany; 3 Chair of Chemical Process Engineering, RWTH Aachen University, Forckenbeckstrasse 51, 52074 Aachen, Germany; 4 Toxicology Centre, University of Saskatchewan, 44 Campus Drive, Saskatoon, SK S7N 5B3 Canada; 5 School of the Environment & Sustainability, University of Saskatchewan, 44 Campus Drive, Saskatoon, SK S7N 5B3 Canada; 6 Department of Geography, RWTH Aachen University, Templergraben 55, 52056 Aachen, Germany; 7 Institute of Sociology, RWTH Aachen University, Eilfschornsteinstrasse 7, 52062 Aachen, Germany; 8 Chair of Management Accounting, RWTH Aachen University, Templergraben 64, 52062 Aachen, Germany; 9 Institute of Neotectonics and Natural Hazards, RWTH Aachen University, Lochnerstrasse 4-20, 52056 Aachen, Germany; 10 Institute of Geology and Geochemistry of Petroleum and Coal, RWTH Aachen University, Lochnerstrasse 4-20, 52056 Aachen, Germany; 11 Canadian Rivers Institute and Biology Department, University of New Brunswick, 100 Tucker Park Road, Saint John, NB E2L 4L5 Canada; 12 Department of Effect-directed Analysis, Helmholtz Centre for Environmental Research UFZ, Leipzig, Saxony Germany; 13 Department of Bioanalytical Ecotoxicology, Helmholtz Centre for Environmental Research UFZ, Leipzig, Saxony Germany

**Keywords:** Flood event, Sediment mobilization, Erosion, Fish exposure, Biomarker, Sediment toxicity, Renaturation, Emergency drinking water treatment, (micro)pollutants removal

## Abstract

Protecting our water resources in terms of quality and quantity is considered one of the big challenges of the twenty-first century, which requires global and multidisciplinary solutions. A specific threat to water resources, in particular, is the increased occurrence and frequency of flood events due to climate change which has significant environmental and socioeconomic impacts. In addition to climate change, flooding (or subsequent erosion and run-off) may be exacerbated by, or result from, land use activities, obstruction of waterways, or urbanization of floodplains, as well as mining and other anthropogenic activities that alter natural flow regimes. Climate change and other anthropogenic induced flood events threaten the quantity of water as well as the quality of ecosystems and associated aquatic life. The quality of water can be significantly reduced through the unintentional distribution of pollutants, damage of infrastructure, and distribution of sediments and suspended materials during flood events. To understand and predict how flood events and associated distribution of pollutants may impact ecosystem and human health, as well as infrastructure, large-scale interdisciplinary collaborative efforts are required, which involve ecotoxicologists, hydrologists, chemists, geoscientists, water engineers, and socioeconomists. The research network “project house water” consists of a number of experts from a wide range of disciplines and was established to improve our current understanding of flood events and associated societal and environmental impacts. The concept of project house and similar seed fund and boost fund projects was established by the RWTH Aachen University within the framework of the German excellence initiative with support of the German research foundation (DFG) to promote and fund interdisciplinary research projects and provide a platform for scientists to collaborate on innovative, challenging research. Project house water consists of six proof-of-concept studies in very diverse and interdisciplinary areas of research (ecotoxicology, water, and chemical process engineering, geography, sociology, economy). The goal is to promote and foster high-quality research in the areas of water research and flood-risk assessments that combine and build off-laboratory experiments with modeling, monitoring, and surveys, as well as the use of applied methods and techniques across a variety of disciplines.

## Background

Safeguarding the availability and quality of water is one of the great scientific, technological, and social challenges of this century, both regionally and globally. In particular, flood events are a major threat to wildlife, human health, agriculture, as well as communal and industrial infrastructure in affected areas. Major flood events that have had detrimental effects to inhabitants and the surrounding environment in flood-prone areas have been well documented in many areas worldwide (e.g., Yamuna River, India; Rhine and Elbe Rivers, Germany; Bow and Red Rivers, Canada; Yangtze River, China; etc.) [1, 2]. In particular, due to heavy rainfalls during the spring of 2013, an enormous amount of 22.75 cubic kilometers of water was introduced into the catchment areas of the Elbe and Danube Rivers, Germany, which corresponded to a surface area of approximately 150 by 150 km at a water depth of one meter [3]. This quantity of water resulted in a 100-year flood in some parts of the Elbe and the Danube Rivers [4].

Statistically, 100-year floods can actually occur several times in a decade due to the uncertainty and variability in the actual intervals between floods. Therefore, it is not unreasonable to have high water levels such as those observed in the Elbe and the Danube River in 1999, 2002, and 2013 occur over such a short time-span. In Germany alone, the total number of deaths resulting from the 2002 and 2013 floods was greater than 30, and the direct economic losses estimated for each flood were between 6 and 11 billion € [5]. The increasing frequency of such extreme flooding events in some areas has led to the development of frameworks and measures for preventive flood protection and flood-risk management [5–7]. The political relevance for Europe is reflected in several European Union and the United Nations directives and activities, each of which is subject to significant research and development requirements [5, 6, 8].

In general, an intensified global water cycle is expected worldwide, which would also lead to an increase in flood risks [9]. The direct role that climate change may play in causing extreme events such as 100-year floods has not yet been conclusively demonstrated [10]. However, a number of anthropogenic activities have significantly contributed to the increase or exacerbation of the intensity and frequency of floods worldwide through the alteration of natural river channels, the habitation of floodplain areas, changes in land use that can increase water runoff, and erosion during heavy rainfall events [6, 11]. Furthermore, the increase in economic value of floodplain areas (e.g., urbanization) leads to an increase in the expected damage caused by flood events. The processes or results of shore erosions and collapses from flood events can impact valuable arable land, roads, buildings, and other anthropogenic activities. In addition, eroded materials associated with flood events can be deposited such that they limit human activities (e.g., ports, waterways). Impairment of water quality can also occur with flood events through exceedances or damage to drinking and wastewater-treatment plants that may promote the growth of harmful microbial communities that directly impair the water supplies to dependent communities.

**Fig. 1:**
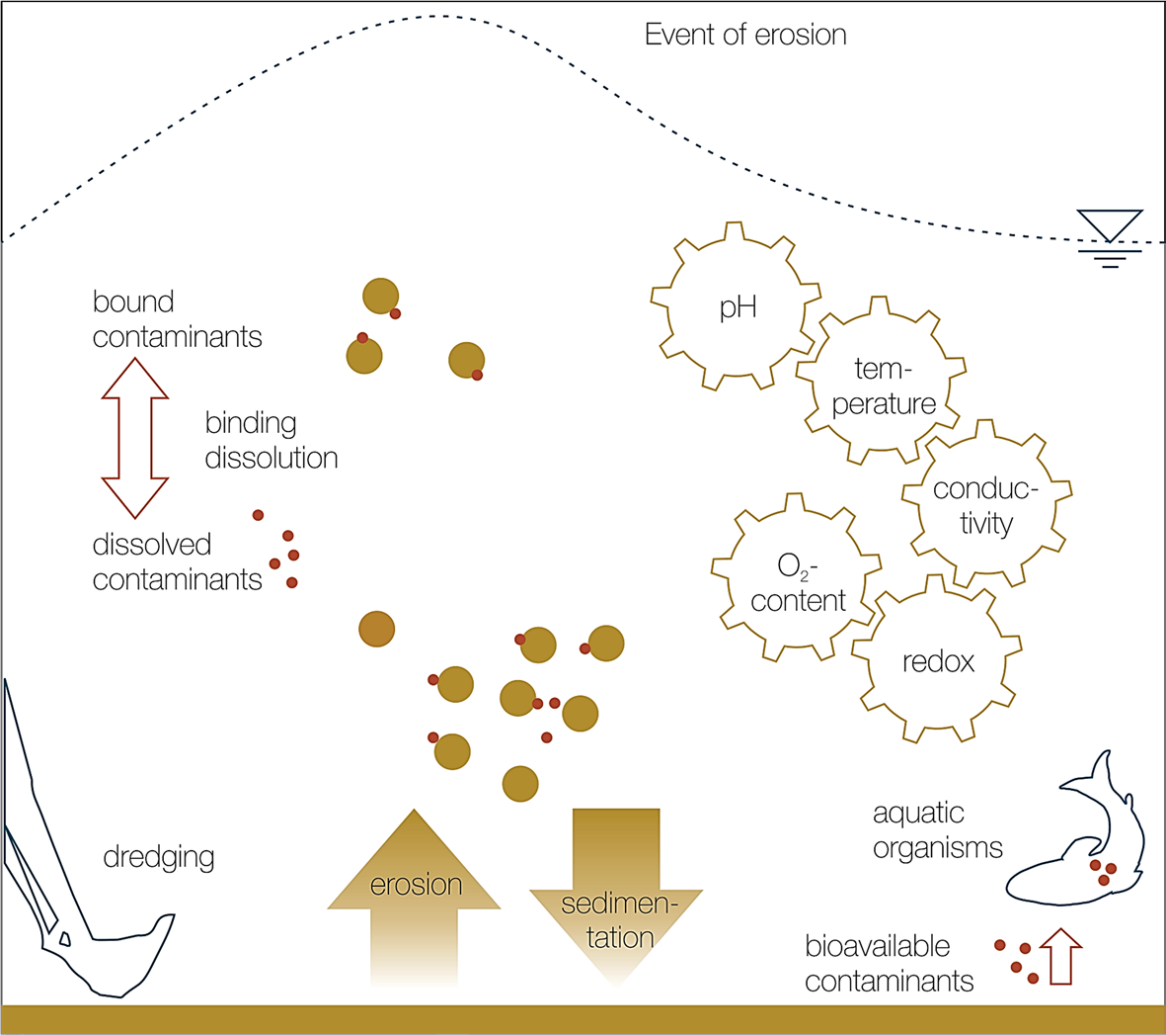
The input and distribution of sediment-associated contaminants in an aquatic system (adapted from [12])

The occurrence of flood events has an impact not only on anthropogenic activities, but also on the hydrodynamic and morphodynamic processes in the water, with effects on flora and fauna, water quality, water and sediment structures, and geomorphology. While it is recognized that flooding is a natural process that can rejuvenate and benefit many rivers and floodplain areas, flooding can also lead to the direct displacement and disruption of aquatic life. In addition, due to the high flow velocities and the resulting bed shear stress, erosive processes can occur in the water of the rivers and along the shores of waterbodies. As a result, sediments enter the water column and are transported in the water as suspended material and/or can settle as bottom sediment (sedimentation). Transport and mobilization of sediments during flood events are an emerging concern as sediments are commonly recognized as both sources and sinks of pollutants in waterbodies. Processes such as dredging, bioturbation, and events of erosion can increase the bioavailability of sediment-bound contaminants to aquatic organisms (Fig. 1 adapted from [12]). The remobilization and distribution of sediment-associated contaminants can impact water quality and the sustainability of aquatic systems, endangering the health of aquatic organisms and wildlife in receiving environments, as well as directly or indirectly affecting human health [13–17]. The European union water framework directive (EU-WFD) has only recently begun to consider the role of sediment-associated contaminants from such long-term sink–sources and potential remobilization, but the breadth of contaminants and the way in which they are considered are still limited [8, 18, 19].

## Aims of project house water

The aim of project house water is to investigate the environmental and socioeconomic impacts and risks of sediment-associated contaminants distributed during and after flood events through close co-operation between ecotoxicologists, hydrologists, water engineers, chemists, geoscientists, water economists, and sociologists to develop new interdisciplinary assessment and management strategies for the distribution of contaminated sediments during flood events. Pollutants enter the aquatic environment and the water cycle from a variety of anthropogenic sources, where they are transported, bound, deposited, and re-released (pathway) or interact with humans, animals, and plants (receptors). A source-pathway-receptor-evaluation allows for technological developments in order to remediate and manage released or bound substances during and after flood events. An integrated water resource management strategy is needed not only to maintain long-term sustainable water quality for both ecosystem and human health but to also protect one of our most important resources—water. The interdisciplinary collaboration of project house water will (i) provide more detailed information on the historic, current, and future sources, and sinks of contaminants in surface waters and sediments; (ii) provide insight into the processes affecting erosion, resuspension, and transport mechanisms of contaminated sediment during flood events; (iii) investigate the bioavailability and effects of contaminated sediments remobilization during flood events; (iv) assess and develop innovative and advanced technologies for the treatment of water affected during flood events; (v) establish a novel methodology for the evaluation of societal impacts from the distribution of contaminants during flood events and subsequent risk communication efforts; and (vi) determine the economic costs associated with societal and environmental impacts from flood events to contribute to a systematic improvement of surface waters within the context of the EU-WFD. These objectives will be achieved through the implementation of six “proof-of-concept” studies outlined later, which will be structured in such a way that researchers from different disciplines work together closely to attain the deliverables.

## Structure of the research in project house water

**Fig. 2:**
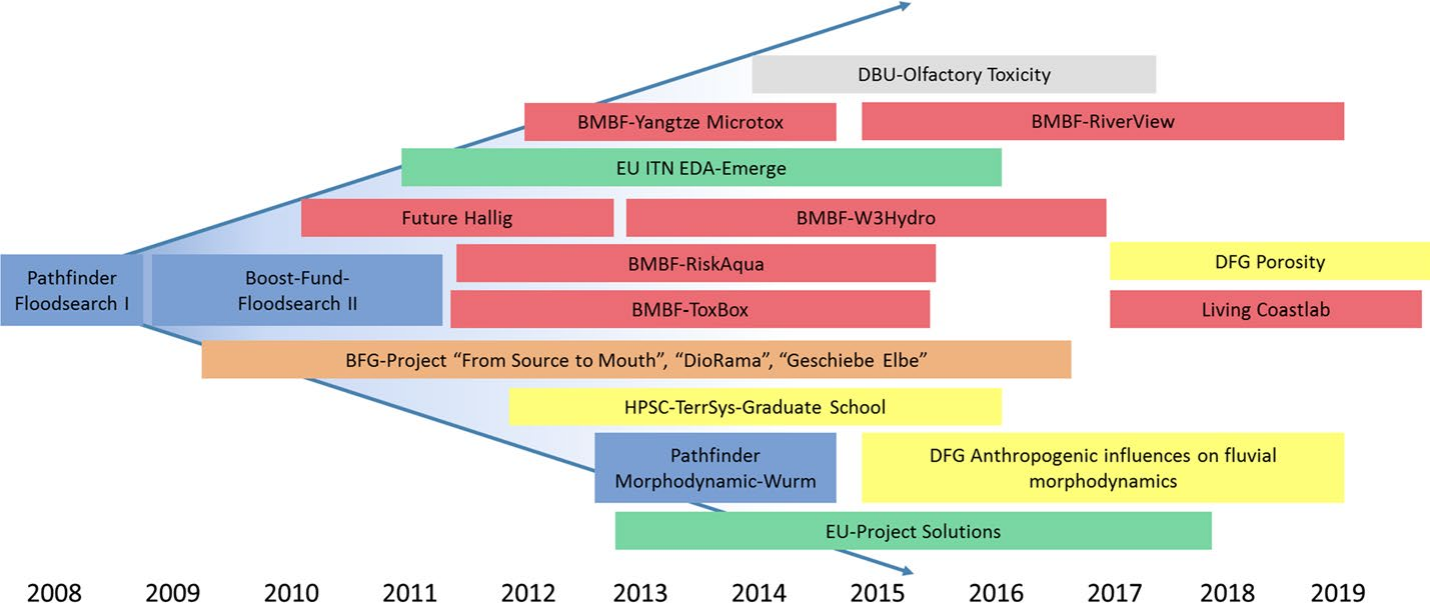
History of water-related research development and previously approved and submitted projects from which project house water has emerged, beginning with the investigations in the Pathfinder/Seed funded project Floodsearch I. *Blue-colored projects* are funded by RWTH Aachen University Exploratory Research Space (ERS) with support from the Deutsche Forschungsgemeinschaft (DFG; German Research Foundation) Excellence Initiative; *orange-colored projects* are funded by the Bundesanstalt für Gewässerkunde (BfG; German Federal Institute of Hydrology); *red-colored projects* are funded by the Bundesministerium für Bildung und Forschung (BMBF; Federal Ministry of Education and Research); *yellow-colored projects* are funded by the DFG (e.g., high-performance scientific computing in terrestrial systems (HPSC) graduate school; *green-colored projects* such as solutions and the European innovative training network effect-directed analysis (EU ITN EDA) Emerge are funded by the European Union; and *gray-colored projects* are funded by the Deutsche Bundesstiftung Umwelt (DBU; German federal foundation for the environment)

**Fig. 3:**
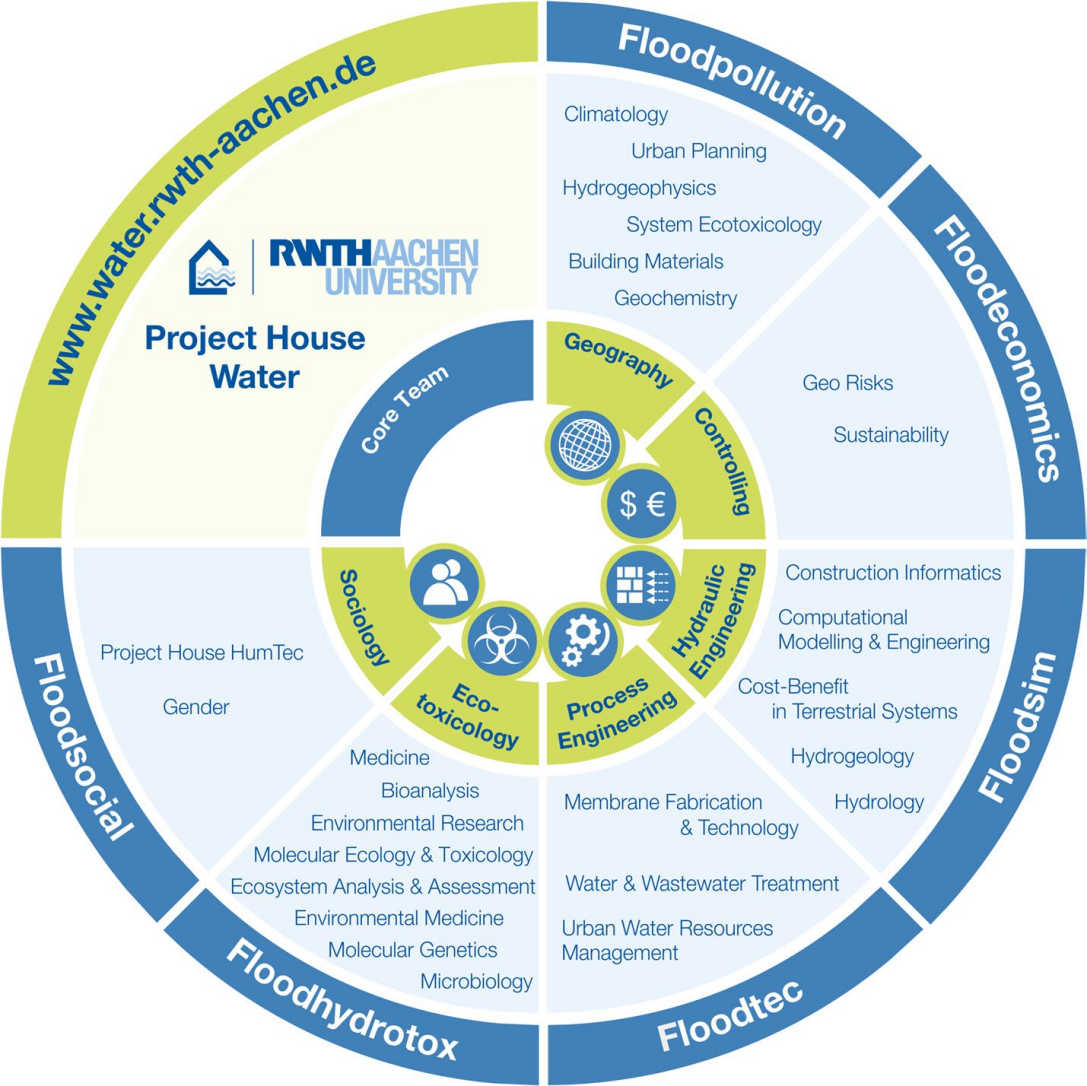
Summary of the interdisciplinary partnerships of project house water with six core teams from Germany and abroad working together on the six proof-of-concept subprojects

The concept of “project houses” was established by RWTH Aachen University, Germany within the framework of the German Excellence Initiative with support of the German research foundation (Deutsche Forschungsgemeinschaft; DFG) to promote and fund interdisciplinary research projects and provide a platform for scientists to collaborate on innovative, challenging research. Project house water was founded from the success of previous exploratory research space pathfinder/seed fund projects and boost-fund projects at the RWTH Aachen University that investigated water-related research (i.e., FLOODSEARCH, DioRAMA, ToxBox; Fig. 2; [8, 20–27]). Project house water promotes the interdisciplinary collaboration of scientists conducting innovative and novel research in the areas of flood research and water research in general. The project house water network is composed of faculties from several institutes at the RWTH Aachen University, and experts from both national and international groups representing several specialized disciplines, working together toward a better understanding of the risk and distribution of contaminants associated with flood events (core team; Fig. 3). The bridging between natural sciences, engineering, and socioeconomics is crucial.

Project house water is structured such that it combines strategically important fields of water research into an interfaculty research network, which serves as the starting point for further areas of related investigation. These fields include water engineering, ecotoxicology, chemistry, geography, chemical process engineering, economics, and sociology. The research conducted under this Project House will consist of individual “proof-of-concept” studies in the area of flood assessment and flood countermeasures, which are briefly outlined later and that will be completed between 2016 and 2019. These subprojects (Fig. 3) integrate technological, social, and economic aspects with sedimentological, morphodynamic and ecotoxicological studies to investigate environmental and socioeconomic impacts of flood events. Project house water promotes interdisciplinarity throughout all aspects of the studies, especially with regard to integrated water assessment in relation to flood risk, sediment dynamics, ecotoxicology, society, health, and global change.

### Flood-pollution: definition of paleo-reference state with regard to geogenic and anthropogenic pollutant propagation through flood events

The reconstruction and process-oriented inventory of historical land use, geological, morphological, and ecotoxicological scenarios of river systems, their floodplains, and their surroundings are important for understanding the consequences of recent and historical flood events and to evaluate and predict future scenarios [28, 29]. Despite recent improvements in the characterization of the paleo-reference state of some rivers (i.e., the state of these systems prior to the introduction of anthropogenic impacts and pollutants; [30–32]), it is still unknown why restoration of river systems is often not successful even through the use of technical, cost-intensive renaturation measures. The subproject “flood-pollution” will investigate difficulties associated with restoring river systems to their paleo-reference conditions and provide guidelines for improvement of restoration efforts. For this reason, sources and sinks of contaminated sediments will be determined and evaluated in selected river systems in Germany. Various project-related and adapted techniques, as well as methods that include historical studies, remote sensing, fieldwork, mapping, sediment sampling in combination with subsequent sedimentological, geochemical, and ecotoxicological sediment analyses, will be used. By comparing characteristics of different fluvial sediments, such as recent flood deposits or historical sediments, and by monitoring pollutants, the influence of sediment contamination is validated. In cooperation with scientists from the other subprojects, river sediments will be sampled and analyzed in regards to different forcing mechanisms. The goal of the subproject “flood-pollution” is to provide recommendations for the integration of the paleo-status and the identification of reference situations of river systems into the EU-WFD.

### Flood-sim: scientific computing of contaminated sediment remobilization during flood events

**Fig. 4:**
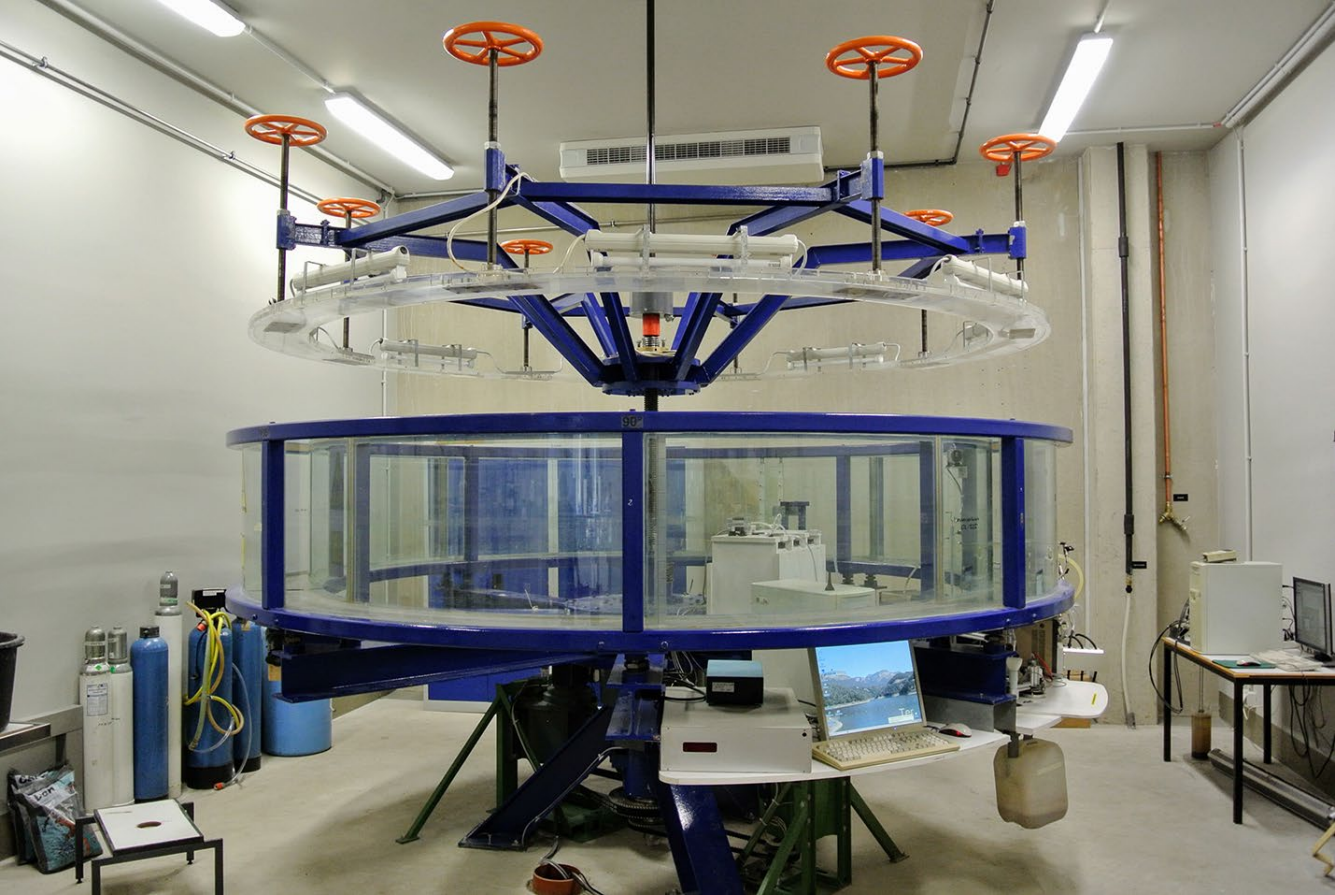
Annular flume at the institute for hydraulic engineering and water resources management, RWTH Aachen University, Germany

Flood events are characterized by turbulent, strongly unsteady, hydrodynamic, sedimentological, morphodynamic and ecotoxicological processes, the description of which is currently not possible in its entirety. Processes and factors governing cohesive sediment erosion and transport, which are particularly responsible for sediment-bound contaminant remobilization, are also not well understood. At the hydraulic laboratory of the Institute of hydraulic engineering and water resources management (RWTH Aachen University, Germany) a series of annular flume (Fig. 4) experiments were previously conducted for the integrated description of the interactions of flood events and associated transport of pollutants. In these experiments, the resuspension of contaminated sediments and resulting ecotoxicological effects were investigated. The goal of “flood-sim” is to build on this earlier work and to numerically describe these previous experiments conducted in the annular flume. Therefore, we aim to implement particle simulations based on a coupled Lattice Boltzmann Method–Discrete Element Method solver to simulate cohesive sediment and contaminant transport. In these simulations, cohesive forces, floc-aggregation and break-up mechanisms, different grain sizes, and forms shall be considered among other factors. The simulations will be validated using experimental data from annular flume experiments. Through interdisciplinary studies and communication with researchers from different disciplines, such as biologists, geographers, and process engineers, we aim to gain more knowledge about processes and factors that affect cohesive sediment resuspension and contaminant release.

Through scientific computing, development of new numerical tools, and usage of high performance computers, such as the JUQUEEN (FZ Jülich; supercomputer; IBM Blue Gene/Q^®^; [33]), the understanding of factors, processes, and interactions influencing the erosion and resuspension of contaminated cohesive sediments will be improved. A key focus will be on the interactions between hydrodynamics, sediment dynamics, pollutant dynamics, and morphodynamics. The outcomes from this research will include a synthesis document and establishment of an interdisciplinary expert network that will provide clear recommendations for the consideration of floods in context with the development of the good water status according to the EU-WFD.

### Flood-hydrotox: quantitative description and prediction of the effects of sediment-bound emerging contaminants on aquatic organisms

In recent years, contaminants of emerging concern such as personal care products and pharmaceuticals (PPCPs), nanoparticles, and flame retardants have gained interest of the public and scientific community, especially with regard to their potential adverse effects on wildlife. One group of chemicals of emerging concern, which has been of particular interest are endocrine-disrupting compounds (EDCs). EDCs are substances that cause disturbances of the hormone systems in wildlife and even humans, potentially impairing reproduction, normal development, growth, and other critical biological functions. Consequently, EDCs have recently been included in the EU-WFD as a group of priority pollutants [34]. An EDC of particular interest is ethinylestradiol (EE2), which is the active ingredient in many birth control pills and has strong estrogenic activity. Recently, it was shown that exposure to environmentally relevant concentrations of EE2 can lead to the collapse of entire fish populations [35]. Due to the hydrophobic nature and the associated octanol–water partition coefficients (log *K*_ow_) of EE2 and other organic EDCs, the potential for sorption to sediment, particularly those with high organic carbon, is great [36–39]. Sediments across Europe, including Germany, Italy, United Kingdom, and the Netherlands revealed endocrine activity from in vitro bioassays such as the yeast estrogen screen (YES) or the chemical-activated luciferase gene expression (CALUX) assay. Estradiol equivalents (EEQs) varied from 0.2 to 1.3 ng/g [40, 41]. Buchinger et al. [37] found elevated concentrations of EDCs such as 4-iso nonylphenols, estrone, and 17β-estradiol in sediment samples from the Luppe River, in the area of Leipzig, Germany compared with ECD concentrations found in sediments from other sampling sites along this river system and other European rivers. Although adverse impacts of EDCs on fish reproduction are well documented and EDCs have been proven to accumulate in the sediment, it is largely unknown to which extent the sediment-bound fraction of EDCs can be remobilized during flood events and hence, to have the potential to cause adverse effects on aquatic organisms. Therefore, the bioavailability of several EDCs (i.e., estrone (E1), ethinylestradiol (EE2), estradiol (E2), and nonylphenols) remobilized from sediments during flood-like conditions and their effects on fish will be investigated in the subproject “flood-hydrotox.” For “flood-hydrotox,” sediment was sampled in 2016 from the Luppe River due to the high concentrations reported by Buchinger et al. [37]. Fish will be exposed in specifically designed systems that allow for the realistic simulation of suspended sediment exposure conditions due to flood events (e.g., annular flume; Fig. 4; [20]). These fish exposures will be conducted in close cooperation with two project partners: the departments of biology and water engineering. This subproject will quantify the remobilization and bioavailability of EDCs from sediments and subsequent effects to fish under flood-like scenarios in order to better aid in the incorporation of such effects from sediment-associated EDCs in the EU-WFD.

### Flood-tec: development of advanced water-treatment technologies addressing flood events

The high water flows associated with flood events often lead to exceedance of the capacity of municipal wastewater-treatment plants (WWTPs), including their hydraulic performance limits and their nutrient- and contaminant-removal capacities. The hydraulic performance of WWTPs directly affects the level of pathogen removal and also the removal of suspended solids and biochemical oxygen demand. As a result of these capacity and performance challenges, untreated pollutants can be discharged into waters in sensitive areas (e.g., drinking water-production areas, recreational waters, etc.), which can pose significant risks to public health and the environment. In cases of flooding, waterbodies that serve as drinking water resources are also likely to receive additional increased loads of suspended solids and pollutants from sources such as pesticide runoff from agricultural land. Effective removal of these pollutants can be difficult with existing water-treatment facilities. The investigations in this subproject “flood-tec” are primarily focused on the further development and adaptation of advanced water-treatment technologies, which can be used in a targeted manner to combat the pollution of flood events. Advanced oxidation processes that are capable of efficiently and rapidly reducing organic and oxidizable inorganic constituents in wastewater are considered. For decentralized drinking water production from contaminated sources, combinations of membrane filtration and adsorption technologies will be examined in this subproject. Porous membranes effectively remove suspended matter and turbidity independent of the raw water quality, whereas adsorption can remove dissolved (micro)pollutants at low cost with high process stability. In particular, alternative membrane-cleaning methods (i.e., temperature-enhanced backwash), as well as adsorbent regeneration pathways (i.e., temperature swing adsorption in liquid phase) have been investigated and will be further developed in this subproject. These two concepts will be implemented together and tested on a laboratory scale and thereafter in the field. Furthermore, emerging electrochemical methods that form hydroxyl radicals by means of a gas diffusion electrode made of carbon nanotubes (CNT) hollow fibers or a boron-doped diamond electrode are also capable of reducing micropollutant contamination [42, 43]. These advanced oxidation processes are particularly suited to eliminate persistent, toxic or nonbiodegradable pollutants. In order to evaluate the innovativeness and societal impact of the investigated technologies, the use of electrochemical methods will also be analyzed from ecotoxicological and socioeconomic points of view. Ecotoxicological quantification of treatment efficiency will supplement chemical water analyses (cf. “flood-hydrotox”). Socioeconomic analysis seeks to evaluate, on the one hand, technology acceptance by a scenario community in a rural setting (cf. “flood-social”) and, on the other hand, economic feasibility including business model development and project cost accounting (cf. “flood-economics”). The goal of this interdisciplinary work is to provide clear recommendations for the utilization of advanced water-treatment technologies required during flood events to achieve good ecological and biological statuses of water according to the EU-WFD and for the protection of public health.

### Flood-social: sociohydrology, flood risk, and crisis communication

New advances in water resources research view water systems like rivers or river deltas as complex sociophysical systems, where the interaction between the humans using and settling the water system and physical dynamics, such as floods or climate change, should be the focus of attention for assessing flood risks [44–46]. From the sociological point of view and in the context of sociohydrology, it is interesting to understand how human behavior affects events like floods. In the “flood-social” subproject, we are especially interested in the social memory and coordination effects of new communication media like Twitter and Facebook. Most pertinent in flood scenarios are two interaction effects impacting the social memory function: the “adaptation effect” [47] and the “levee effect” [48, 49]. These effects can be analyzed in greater detail because they are based on information diffusion, information availability, and information recency, and these features depend not only on the frequency and strength of flood events, but also on the communication technologies used in spreading and preserving information. Studies from Australia suggest that Twitter may be a useful instrument to spread vital information fast and localized in a flood crisis [50]. The aim of the “flood-social” subproject is to evaluate the impacts of different social memory functions on the “levee” and the “adaptation effect” of communities and investigate how local levels of awareness are shaped by communication technologies in the event of a flood crisis.

Research questions of interest include (1) is communication technology such as Twitter a proper tool to relate location-specific information in the case of a flood crisis?; (2) what impacts the social memory function of a river community besides frequency and strength of flooding events?; (3) can we identify styles of more technological-oriented and more sustainable-oriented river communities?; (4) what types of communication technologies were used in a case of a flood crisis and which were used by the institutional actors?; (5) how does the overall awareness of risks concern flood events in river communities?; and (6) are there measures to raise awareness on the risks involved in flood events? Our expertise resides in measuring the impact of communication technologies in different areas of society and in sociotechnical or sociophysical systems, analyzing human behavior patterns related to sociophysical environments, analyzing networks of on- and offline and generating data on the cultural preferences of communities regarding technological or material risks. Therefore, the subproject “flood-social” will work closely with the other research partners to identify river communities of interest to investigate the abovementioned questions.

### Flood-economics: economic assessment of pollutant propagation after flood events

The distribution of pollutants after flood events has an economic component in addition to social and ecological impacts. Economic consequences can be roughly differentiated in terms of direct (e.g., demolished infrastructure) and indirect (e.g., ecotoxicological and human adverse effects) costs. These consequences can translate into different types of costs, which reflect the action required to respond to potential environmental impacts. Prevention and protection costs reflect the economic consequences to fully or partially avoid negative environmental impacts and include costs of reducing the pollution of harmful substances in the environment as a result of flood events. The calculations in this subproject are mainly focused on the costs of the consequences of harmful emissions of pollutants associated with flood events. Since the dispersion of pollutants are subject to numerous factors (i.e., flood development, existing pollutant sources and sinks, hydro- and morphodynamics, flood-protection measures, population of flora and fauna, natural adaptability of ecosystems, proximity to drinking water-protection areas), environmental and economic impacts need to be estimated on the basis of scenario-based simulation studies. Calculating the distribution of harmful substances in ecosystems allows for an economic assessment of flood events, the consequences of which should consider the resulting damage and subsequent repair of ecosystems. Utilizing information obtained from other subprojects regarding their social and environmental assessments, the goal of this subproject is to derive an economic framework for the quantification of costs associated with prevention and elimination of flood-related impacts. The consideration of noneconomic parameters from other subprojects provides the unique opportunity to quantify this framework using specific measurements. For example, in collaboration with the “flood-tec” subproject, we can derive not only cost structures for novel water-treatment technologies, but also provide a sustainable business model assessing the prevention of the current monetary threat of flood events and its subsequent negative ecological impact. In addition, the negative ecological impact associated with the occurrence of EDC compounds in sediments examined by the “flood-hydrotox” subproject can be transformed into monetary variables, feeding the simulation models.

## Success and long-term significance of project house water

The combination of biological, chemical, hydrological, toxicological, geographical, technological, social, and economic disciplines in project house water, which are traditionally considered independently, allows for highly innovative assessments of the risk and consequences for human health and the states of ecosystems from exposure to contaminants and other stressors associated with flood events. The proposed research of the subprojects represents an important step toward a novel interdisciplinary investigative approach of pollutant transport in surface waters in the context of the EU-WFD. The interdisciplinary cooperation is an integral feature of the subprojects, which allows for the identification of measures and policies to reduce of the economic, ecological, and social consequences from flood events and is also of great importance for follow-up applications and the development of new methods that build on project house water research. Interdisciplinary collaboration of researchers to address a mutual research question is not a new concept, but it is unfortunately not always utilized. To overcome challenges of interdisciplinary research (i.e., lack of historical interdisciplinary cooperation, extra time requirements, differences in methodologies; [51]), the concepts, themes, questions, and research designs addressed in this research were mutually developed and are conducted by researchers from multiple disciplines. In addition, regular communication and frequent coordination of team members occur throughout the project, and ultimately the results and knowledge produced from this collaboration will provide feedback and the building blocks for future interdisciplinary research questions. The long-term goal of project house water is to continue to foster high-quality research in the fields of water research and flood-risk assessments, with the potential for collaboration, not only within the RWTH Aachen University but also with the numerous national and international colleagues in various academia, government, and industrial institutes. This work has been and, will continue to be, promoted at local, national, and international platforms to attract and further expand upon the existing research and scientific network of flood-related risk assessment. With promotion of this project house water research network early on, we hope to create a dialog with other researchers in the scientific community, who may be conducting similar works and to increase communication so that complementary investigations and research can be fostered to broaden the research network and efficiency of data output and sharing of data.

## Abbreviations

BfG: Bundesanstalt für Gewässerkunde (German federal institute of hydrology)
BMBF: Bundesministerium für Bildung und Forschung (German federal ministry of education and research)
CNT: carbon nanotubes
DFG: Deutsche Forschungsgemeinschaft (German research foundation)
DBU: Deutsche Bundesstiftung Umwelt (German federal foundation for the environment)
ERS: exploratory research space
EU ITN EDA: European union innovative training network effect-directed analysis
FZJ: Forschungszentrum Jülich (research center Jülich, Germany)
HPSC: high-performance scientific computing in terrestrial systems
JUQUEEN: Jülich BlueGene/Q Supercomputer
RWTH: Rheinisch-Westfälische Technische Hochschule
UFZ: Helmholtz centre for environmental research
WFD: water framework directive
WWTPs: wastewater-treatment plants
